# ATP synthase subunit-β down-regulation aggravates diabetic nephropathy

**DOI:** 10.1038/srep14561

**Published:** 2015-10-09

**Authors:** Siao-Syun Guan, Meei-Ling Sheu, Cheng-Tien Wu, Chih-Kang Chiang, Shing-Hwa Liu

**Affiliations:** 1 Institute of Toxicology, College of Medicine, National Taiwan University, Taipei, Taiwan; 2 Institute of Nuclear Energy Research, Atomic Energy Council, Executive Yuan, Taoyuan, Taiwan; 3 Biomedical Sciences, College of Life Sciences, National Chung Hsing University, Taichung, Taiwan; 4 Departments of Integrated Diagnostics & Therapeutics and Internal Medicine, National Taiwan University Hospital and National Taiwan University College of Medicine, Taipei, Taiwan; 5 Department of Medical Research, China Medical University Hospital, China Medical University, Taichung, Taiwan; 6 Department of Pediatrics, National Taiwan University Hospital, Taipei, Taiwan

## Abstract

In this study, we investigated the role of ATP synthase subunit-β (ATP5b) in diabetic nephropathy. Histopathological changes, fibrosis, and protein expressions of α-smooth muscle actin (α-SMA), advanced glycation end-products (AGEs), and ATP5b were obviously observed in the kidneys of *db/db* diabetic mice as compared with the control *db/m*^+^ mice. The increased ATP5b expression was majorly observed in diabetic renal tubules and was notably observed to locate in cytoplasm of tubule cells, but no significant increase of ATP5b in diabetic glomeruli. AGEs significantly increased protein expression of ATP5b and fibrotic factors and decreased ATP content in cultured renal tubular cells via an AGEs-receptor for AGEs (RAGE) axis pathway. Oxidative stress was also induced in diabetic kidneys and AGEs-treated renal tubular cells. The increase of ATP5b and CTGF protein expression in AGEs-treated renal tubular cells was reversed by antioxidant N-acetylcysteine. ATP5b-siRNA transfection augmented the increased protein expression of α-SMA and CTGF and CTGF promoter activity in AGEs-treated renal tubular cells. The *in vivo* ATP5b-siRNA delivery significantly enhanced renal fibrosis and serum creatinine in *db/db* mice with ATP5b down-regulation. These findings suggest that increased ATP5b plays an important adaptive or protective role in decreasing the rate of AGEs-induced renal fibrosis during diabetic condition.

Diabetic nephropathy is a major cause of chronic kidney disease and presents high morbidity and mortality rate in diabetic patients^1^,^2^. The clinical study indicated that incipient nephropathy (microalbuminuria stage) occurs in 20–40% of patients 10–15 years after the onset of diabetes, and overt nephropathy (macroalbuminuria stage) affects 20–40% of patients 15–20 years after the onset of diabetes^3^. Diabetic nephropathy is associated with the development of characteristic histopathological features, including thickening of glomerular and tubular basement membrane, glomerular and tubular hypertrophy, mesangial matrix expansion, and glomerulosclerosis and tubulointerstitial fibrosis^4^,^5^. Diabetic hyperglycemia causes the increase of glucose influx into the kidney and leads to the hemodynamic and metabolic disturbances, which are responsible for the renal pathology^6^. Advanced glycation end-products (AGEs) are produced from non-enzymatic reactions between reducing sugars and amino groups of proteins under hyperglycemic conditions in diabetes mellitus^7^,^8^. Initially, reversible Amadori product is formed, which undergo further complex reaction, including rearrangement, dehydration, and condensation, to produce irreversible AGEs^9^. The formation and accumulation of AGEs are known to conduct the characteristic features in diabetes^10^,^11^,^12^. Previous studies showed that AGEs induced fibrogenesis and inflammatory reactions and consequently contributed to the development of diabetic nephropathy^13^,^14^,^15^,^16^. Although the hemodynamic and metabolic changes by AGEs production under diabetes are defined, the molecular mechanisms of AGEs-related renal fibrosis are not fully understood.

Mitochondrial dysfunction is thought to be a central mediator to reduce cellular ATP synthesis. It has been demonstrated that AGEs impair the mitochondrial function to cause the islet β-cell dysfunction^17^. The expression of ATP synthase subunit-β (ATP5b), a subunit of mitochondrial ATP synthase (complex-V) for ATP biosynthesis, has been shown to be altered in islets of type 2 diabetic rats^18^ and in high glucose-treated renal proximal tubular cells *in vitro*^19^. Moreover, Højlund *et al.* have found that human ATP5b protein presented abnormal phosphorylation in insulin-resistant muscles of obesity and diabetic patients^20^. These empirical results suggested that ATP5b might contribute in the diabetic complications. However, the role of ATP5b in diabetic nephropathy or AGEs-related renal fibrosis remains unclear. Hence, we hypothesized that the expression of ATP5b is altered in the diabetic kidneys and is involved in the AGEs-related renal fibrosis. In this study, we aimed to investigate whether ATP5b plays a role in diabetic nephropathy especially in AGEs-related renal fibrosis *in vivo* and *in vitro*. The present findings demonstrate that ATP5b protein expression is increased and may play a protective role in the kidneys during early diabetic nephropathy.

## Results

### Alterations in renal function and serum biochemical parameters in *db/db* mice

The body weight (37.82 ± 3.01 versus 24.97 ± 2.44 g, *p* < 0.01), fasting plasma glucose (344.08 ± 17.39 versus 109.52 ± 9.36 mg/dl, *p* < 0.01), and serum insulin (5.58 ± 0.79 versus 1.09 ± 0.44 μg/l, *p* < 0.01) in *db/db* mice were significantly increased as compared with control *db/m*^+^ mice. The glycated hemoglobin (HbA1c) levels in *db/db* mice were higher than control *db/m*^+^ mice (7.18 ± 0.23 versus 3.59 ± 0.38%, *p* < 0.01). Furthermore, the creatinine levels in bloods were significantly increased in *db/db* mice as compared with *db/m*^+^ mice (0.69 ± 0.02 versus 0.41 ± 0.01 mg/dl, *p* < 0.01).

### AGEs formation, ATP5b expression, histopathological changes, and fibrosis in kidneys of *db/db* mice and diabetic patients

**Figure 1:**
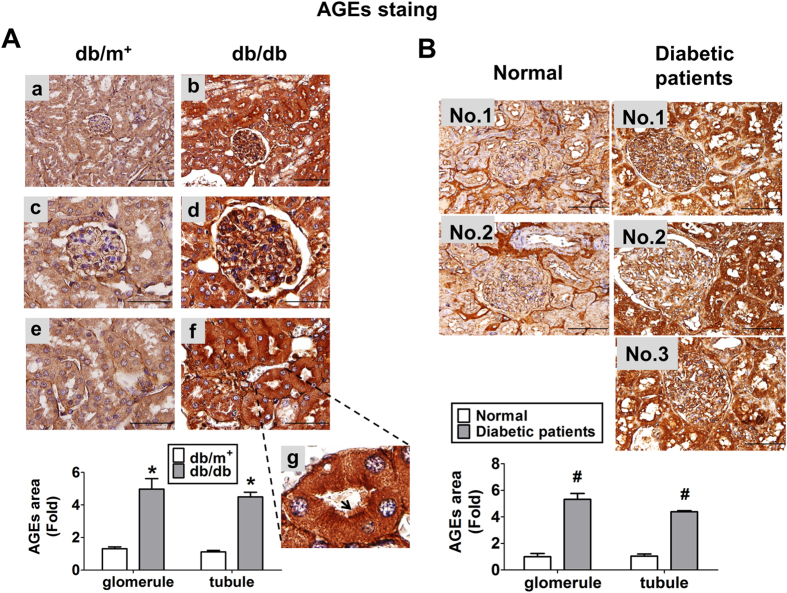
Immunohistochemical staining for AGEs in the kidneys of diabetic *db/db* mice or patients. The immunohistochemical staining for AGEs were performed on the renal sections of *db/db* mice (**A**) or diabetic patients (**B**). In *db/m*^+^ or *db/db* mice, low magnification field of vision were shown as (**A-a**) and (**A-b**); glomeruli were shown in (**A-c**) and (**A-d**); proximal tubules were shown as (**A-e**) and (**A-**f). In (**A-f**), the representative area of brush border in proximal tubule was indicated and its enlarged scale was shown in (**A-g**). In A-g, the arrow indicated the brush border of proximal tubule. Original magnification, ×400, scale bar: 100 μm; ×1000, scale bar: 50 μm. The semi-quantitative assessment of immunohistochemistry with three random areas per section was determined by ImageJ software. Data are presented as means ± SEM (n ≥ 6). *P < 0.05, *db/db* versus *db/m*^+^; #*P* < 0.05, diabetic patients versus normal subjects.

**Figure 2:**
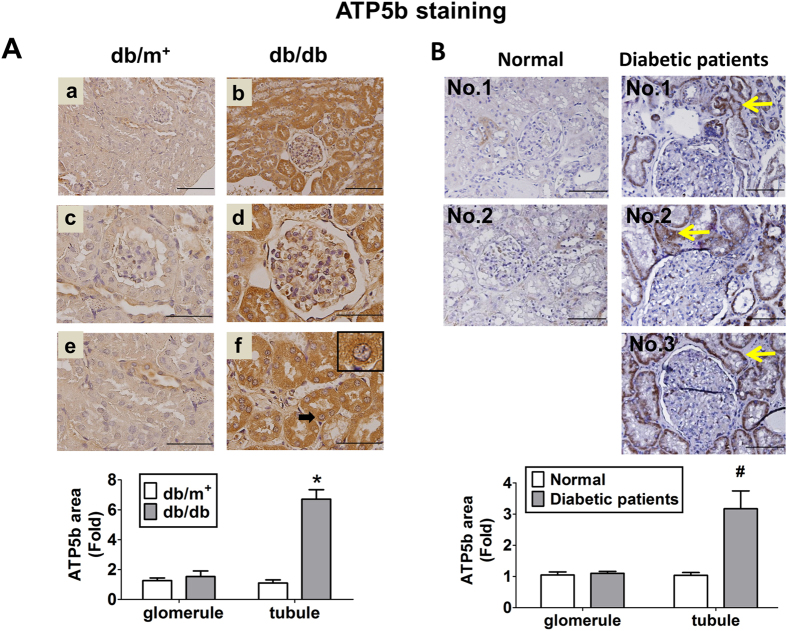
Immunohistochemical staining for ATP5b in the kidneys of diabetic *db/db* mice or patients. The immunohistochemical staining for ATP5b was performed on the renal sections of diabetic *db/db* mice (**A**) or diabetic patients (**B**). In diabetic *db/m*^+^ or *db/db* mice, low magnification field of vision were shown as (**A-a**) and (**A-b**); glomeruli were shown in (**A-c**) and (**A-d**); proximal tubules were shown as (**A-e**) and (**A-f**). Scale bar: 50 μm. In B, the arrow indicated the representative area of ATP5b expression in the renal tissues of diabetic patients. The semi-quantitative assessment of immunohistochemistry with three random areas per section was determined by ImageJ software. Data are presented as means ± SEM (n ≥ 6). *P < 0.05, *db/db* versus *db/m*^+^; #*P* < 0.05, diabetic patients versus normal subjects.

The immunohistochemical staining for AGEs was obviously augmented in the kidneys of *db/db* mice (Fig. 1A) and diabetic patients (Fig. 1B), which AGEs were localized in the renal glomerulus and tubular areas. The immunohistochemical staining for ATP5b in the kidneys of *db/db* mice (Fig. 2A) and diabetic patients (Fig. 2B) were also higher than that in *db/m*^+^ mice and normal subjects, respectively. The increased ATP5b protein expressions were majorly observed in renal tubule areas. The increased ATP5b were notably located in cytoplasm of renal tubule cells under the high power magnification (Fig. 2A-f). However, there was no significant difference in glomerular ATP5b protein expressions between *db/db* and *db/m*^+^ mice, and similarly, between diabetic patients and normal subjects. These results indicated that ATP5b expression was markedly increased in renal tubule area of *db/db* mice, but not in renal glomerulus area. The renal histopathology in *db/db* mice also revealed the thickening of glomerular mesangium and basement membrane, increased vacuole formation of proximal tubules, and contraction of epithelial luminal space (data not shown).

**Figure 3:**
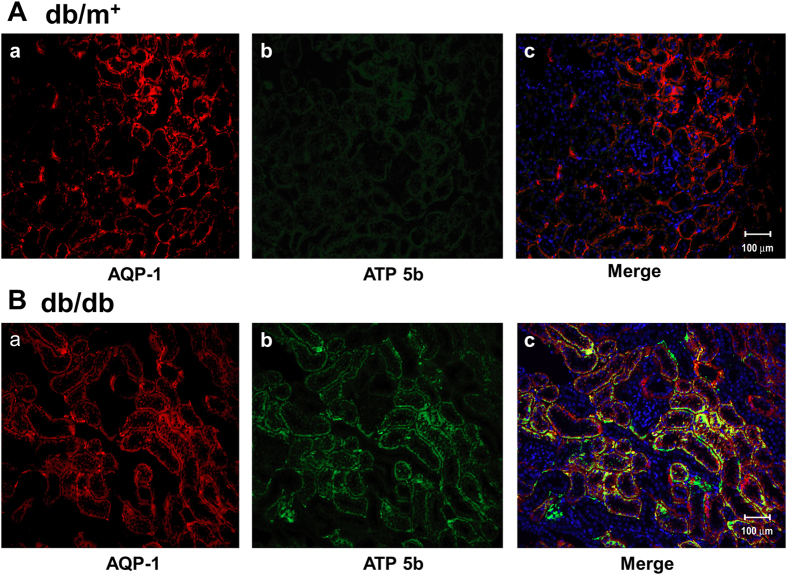
The distribution of ATP5b in the renal proximal tubules of diabetic mice. Double immunofluorescence staining was performed for identifying AQP-1 (red), ATP5b (green), and nuclei (blue) in renal sections from *db/m*^+^ (**A**) and *db/db* (**B**) mice (n = 6). AQP-1 is a renal proximal tubule marker. The ATP5b^+^/AQP-1^+^ tissue was presented as yellow color in the merged image. Hoechst 33258 counter staining was performed for nuclei location (blue color). Representative results from at least three individual experiments are shown. Scale bar: 50 μm.

In order to detect the distribution of ATP5b on renal proximal tubules in *db/db* mice, AQP-1, a renal proximal tubular marker, was performed for double immunofluorescence staining. We observed that the overexpressed ATP5b was significantly located on renal proximal tubules in *db/db* mice (Fig. 3).

**Figure 4:**
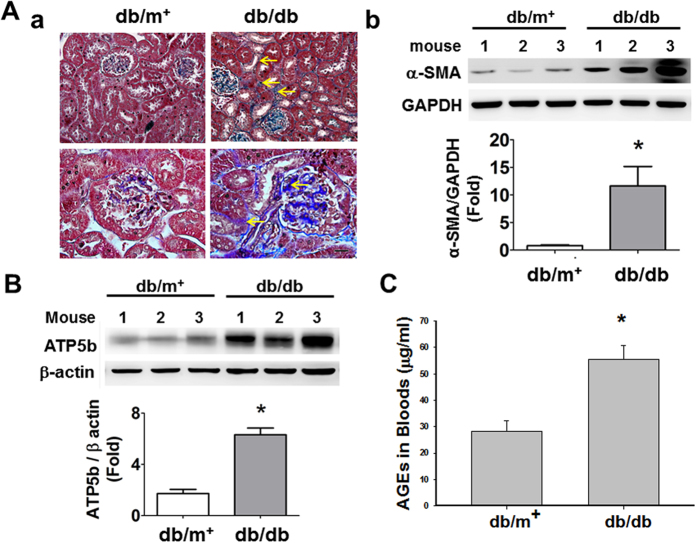
Renal fibrosis and protein expressions of α-SMA, AGEs, and ATP5b in the kidneys of *db/db* and *db/m*^+^ mice. (**A-a**) Masson’s trichrome stains were performed on renal tissue sections of *db/db* or *db/m*^+^ mice. Top panel: ×400, scale bar: 50 μm; bottom panel: ×1000, scale bar: 20 μm. The progression of renal fibrosis, which was shown in glomerular and tubulo-interstitial areas, was significantly increased in the *db/db* mice as compared with *db/m*^+^ mice. The arrow indicated the representative renal fibrosis area in *db/db* mice. The protein expressions of α-SMA (**A-b**) and ATP5b (**B**) were determined by Western blotting. The protein levels of immunoblotting were quantified by densitometry and normalized by GAPDH or β-actin levels. Data are presented as means ± SEM (n ≥ 3). *P < 0.05 *db/db* versus *db/m*^+^. (**C**) The levels of blood AGEs were determined by a mouse ELISA kit. Data are presented as means ± SEM (n = 16 for *db/m*^+^ mice and n = 13 for *db/db* mice). *P < 0.05, *db/db* versus *db/m*^+^ mice.

The progression of renal fibrosis, which was shown in glomerular and tubulointerstitial areas, was severer in *db/db* mice than in *db/m*^+^ mice (Fig. 4A-a). Furthermore, the renal protein expressions of fibrotic marker α-smooth muscle actin (α-SMA, Fig. 4A-b) and ATP5b (Fig. 4B) and the blood levels of AGEs (Fig. 4C) were significantly increased in *db/db* mice.

### *In vitro* effects of AGEs on cell viability, ATP5b expression, and fibrotic signaling molecules

**Figure 5:**
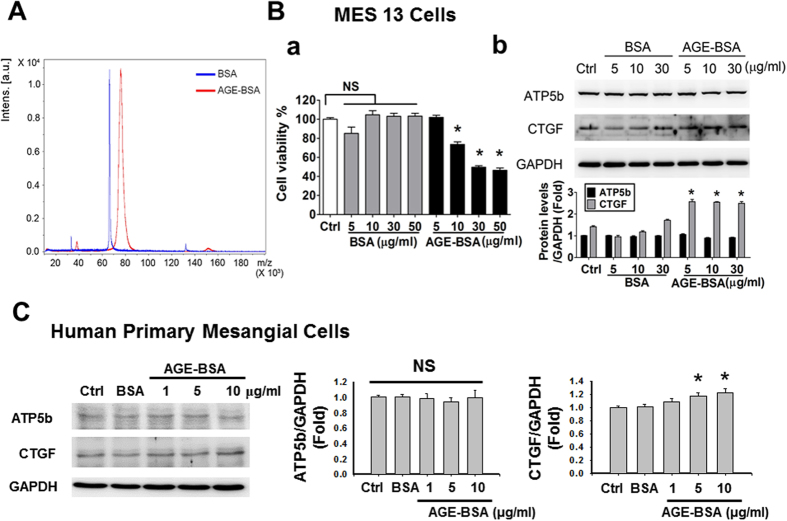
Effects of AGE-BSA on cell viability and protein expressions of ATP5b and CTGF in cultured glomerular mesangial cells. (**A**) AGE-BSA was identified using a MALDI-TOF/TOF mass spectrometry. Mouse mesangial cells (MES13) (**B**) or human primary mesangial cells (**C**) were treated with AGE-BSA or BSA (1–50 μg/ml) for 24 hours. Cell viability was determined by CCK-8 assay (**B-a**). Protein expressions of ATP5b and CTGF in MES13 cells (B-b; AGE-BSA and non-glycated BSA, 5–30 μg/ml) and human primary mesangial cells (**C**; AGE-BSA, 1–10 μg/ml; non-glycated BSA, 10 μg/ml) were determined by Western blotting. Protein levels were quantified by densitometry and normalized by GAPDH levels. Data are presented as means ± SEM (n ≥ 4). *P < 0.05, AGE-BSA versus BSA. NS: non-significant.

**Figure 6:**
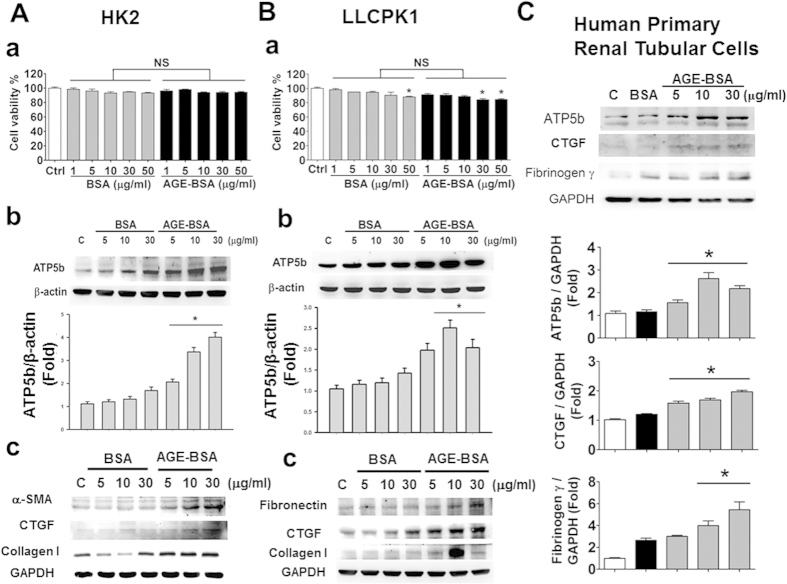
Effects of AGE-BSA on cell viability and protein expressions of ATP5b and fibrotic signaling molecules in cultured renal proximal tubular cells. HK-2 cells (**A**), LLC-PK1 cells (**B**) and human primary renal tubular cells (**C**) were treated with AGE-BSA or BSA (1–50 μg/ml) for 24 hours. Cell viability of HK-2 (**A-a**) and LLCPK1 (B-a) cells was determined by CCK-8 assay. Protein expressions of ATP5b and fibrotic signaling molecules (CTGF, α-SMA, collagen I, fibronectin, and fibrinogen γ) in HK-2 (**A-b,c**), LLCPK1 (**B-b,c**), and human primary renal tubular cells (**C**) were determined by Western blotting. Protein levels were quantified by densitometry and normalized by β-actin or GAPDH levels. Data are presented as means ± SEM (n = 5). *P < 0.05, AGE-BSA versus BSA. NS: non-significant.

Prepared AGE-BSA was identified using a MALDI-TOF/TOF mass spectrometry (Fig. 5A). The concentrations of AGEs at 10–50 μg/ml, but not at 5 μg/ml, significantly decreased the mouse mesangial cell viability (Fig. 5B-a). Both AGEs and non-glycated bovine serum albumin (BSA) with the concentrations of 5–30 μg/ml did not affect the protein expression of ATP5b in mouse mesangial cells (Fig. 5B-b) and human primary mesangial cells (Fig. 5C). However, the connect tissue growth factor (CTGF) protein expression was significantly increased by AGE-BSA treatment in both mouse and human mesangial cells (Fig. 5B-b,C). On the other hand, both AGE-BSA and non-glycated BSA (1–50 μg/ml) did not affect the cell viability in human renal proximal tubular cell line HK-2 (Fig. 6A-a), but slightly reduced the cell viability at higher concentrations in pig renal proximal tubular cell line LLC-PK1 (Fig. 6B-a). However, there was no significant difference of cell viability between AGE-BSA group and nonglycated BSA group in both cell lines. Moreover, AGE-BSA (5–30 μg/ml) markedly and significantly increased the protein expression of ATP5b in HK-2 and LLC-PK1 cells (Fig. 6A-b,B-b) and human primary renal proximal tubular cells (Fig. 6C) in a dose-dependent manner. AGE-BSA (5–30 μg/ml) also significantly increased the protein expressions of α-SMA, fibronectin, fibrinogen γ, CTGF, and collagen-1 in HK-2 (Fig. 6A-c) and LLC-PK1 (Fig. 6B-c) and human primary renal proximal tubular cells (Fig. 6C) in a dose-dependent manner.

**Figure 7:**
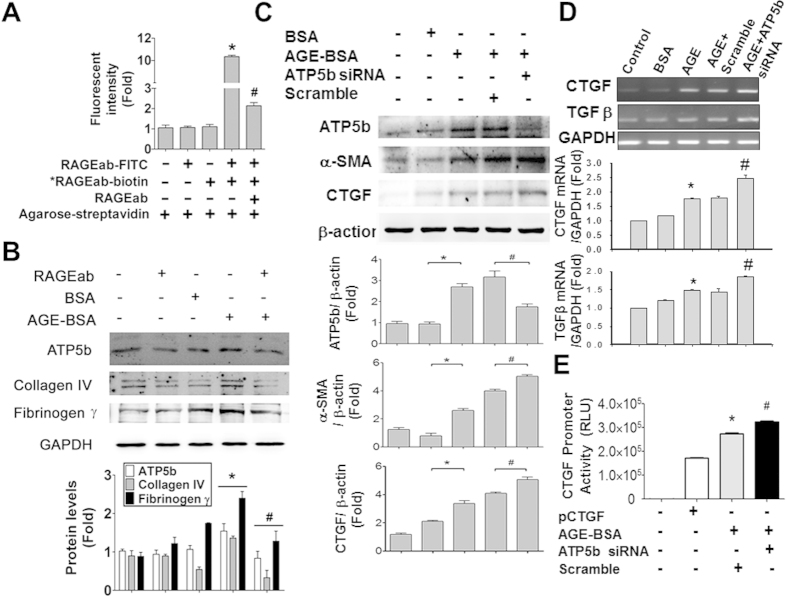
RAGE blocking suppressed and ATP5b knockdown enhanced AGE-BSA-induced expression of fibrotic signaling molecules in cultured renal proximal tubular cells. (**A**) RAGE antibody binding to RAGE proteins assay in renal proximal tubular cells. The HK-2 cells were treated with the FITC-labeled RAGE antibody (RAGEab-FITC, 10 μg/ml) for 1 hour. The ability of RAGE antibody binding to RAGE proteins was detected by fluorescence ELISA reader. *RAGEab-biotin: biotin-conjugated RAGE antibody. Agarose-streptavidin: streptavidin-conjugated agarose beads. (**B**) HK-2 cells were incubated with AGE-BSA (30 μg/ml) for 24 hours with or without RAGE antibodies (RAGEab, 10 μg/ml) pretreatment for 1 hour. (**C**) HK-2 cells were transiently transfected with ATP5b siRNA (80 nM) for 6 hours and subsequently treated with AGE-BSA (30 μg/ml) for 24 hours. Protein expressions of collagen IV, fibrinogen γ, α-SMA, CTGF, and ATP5b were determined by Western blotting (**B**,**C**). Quantification of immunoblotting was determined by densitometric analysis. (**D**) The mRNA expressions of CTGF and TGFβ were determined by real-time PCR. HK-2 cells were exposed to AGEs for 24 hours and then subjected to rela-time PCR assay. The mRNA levels were quantified by densitometry and normalized by *GAPDH* levels. (**E**) The reporter assay for *CTGF* gene. HK-2 cells were exposed to AGEs for 24 hours with or without ATP5b siRNA transfection. Dual luciferase reporter assay was performed to observe the effect of ATP5b on CTGF transcriptional activity. Data are presented as means  ± SEM (n ≥ 5). *P < 0.05, column 4 versus column 3 (**A**); AGE-BSA versus BSA (**B–D**); AGE-BSA/scramble siRNA versus pCTGF vector (**E**). #*P* < 0.05, column 5 versus column 4 (**A**); AGE-BSA/RAGEab versus AGE-BSA (**B**); AGE-BSA/ATP5b siRNA versus AGE-BSA/scramble siRNA (**C-E**).

AGE-receptor for AGE (RAGE) axis has been demonstrated to be involved in the pathogenesis of diabetic nephropathy^21^. We next investigated whether AGE-RAGE axis affects the expressions of ATP5b and fibrogenesis-related proteins in HK-2 cells. The blockade ability of RAGE neutralizing antibody for RAGE-ligand interaction has been shown in the previous studies^22^,^23^. We also confirmed the ability of RAGE antibody binding to RAGE protein in HK-2 cells (Fig. 7A). We further found that the increases of protein expressions of fibrotic markers (collagen IV and fibrinogen γ) and ATP5b by AGE-BSA in HK-2 cells were significantly suppressed by RAGE neutralizing antibody (Fig. 7B).

We next determined the role of ATP5b in AGE-BSA-induced renal fibrosis process. The results indicated that transfection of ATP5b siRNA markedly decreased the ATP5b protein expression and significantly enhanced the protein expressions of α-SMA and CTGF (Fig. 7C) and mRNA expressions of CTGF and TGFβ (Fig. 7D) in AGE-BSA-treated HK-2 cells. Moreover, AGE-BSA significantly increased the CTGF promoter activity in HK-2 cells, which could be significantly enhanced by transfection of ATP5b siRNA (Fig. 7E).

### Role of oxidative stress in the regulation of ATP5b expression by AGE-BSA

**Figure 8:**
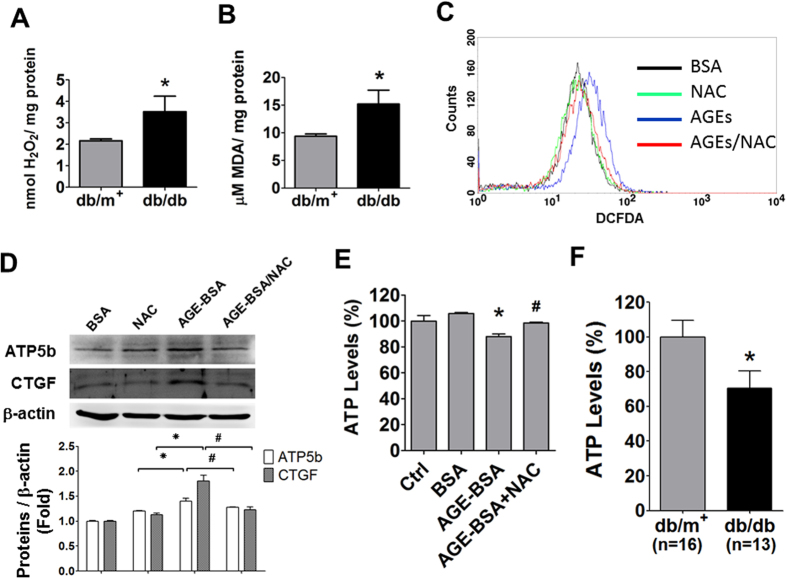
Effects of AGE-BSA on oxidative stress, ATP5b expression, CTGF expression, and ATP production in cultured renal proximal tubular cells. The levels of hydrogen peroxide (**A**) and lipid peroxidation (**B**) in the kidneys of *db/db* and *db/m*^+^ mice were detected. (**C**,**D**) The effects of N-acetylcysteine (NAC) on AGE-BSA treated HK-2 cells. The HK-2 cells were treated with AGE-BSA (30 μg/ml) for 24 hours in the presence or absence of NAC (2 mM). The levels of ROS production was detected by using DCFDA flow cytometry (**C**). The protein expressions of ATP5b and CTGF were measured by Western blotting (**D**). Protein expression was quantificated by densitometry and normalized by β-actin levels. (**E**,**F**) ATP content assay in renal tubular cells and diabetic kidneys. HK-2 cells were treated with AGE-BSA (30 μg/ml) for 24 hours (**E**). The luminescence ATP detection assay was performed for measuring ATP contents in HK-2 cells (**E**) and kidneys of *db/db* and *db/m*^+^ (**F**). Data are presented as means ± SEM (n ≥ 4 for *in vitro* experiments; n = 16 for *db/m*^+^ mice and n = 13 for *db/db* mice). *P < 0.05, AGE-BSA versus BSA; *db/db* versus *db/m*^+^. ^#^*P* < 0.05, AGE-BSA/NAC versus AGE-BSA.

AGE-RAGE axis has been demonstrated to be involved in the reactive oxygen species (ROS) production^24^. We also observed that the levels of hydrogen peroxide and lipid peroxidation were increased in the kidneys of *db/db* mice (Fig. 8A,B). The levels of ROS production were also increased in AGE-BSA-treated HK-2 cells (Fig. 8C). Both increased ATP5b and CTGF protein expressions in AGE-BSA-treated HK-2 cells could be reversed by antioxidant N-acetylcysteine (Fig. 8D). Treatment with AGE-BSA (10 μg/ml) for 24 h slightly but significantly decreased the ATP contents in HK-2 cells as compared with BSA alone (Fig. 8E). The decreased ATP contents by AGE-BSA could be reversed by N-acetylcysteine (Fig. 8E). The ATP contents in the kidneys of *db/db* mice were also significantly decreased as compared with *db/m*^+^ mice (Fig. 8F).

### ATP5b siRNA *in vivo* delivery enhanced renal fibrosis in *db/db* mice

**Figure 9:**
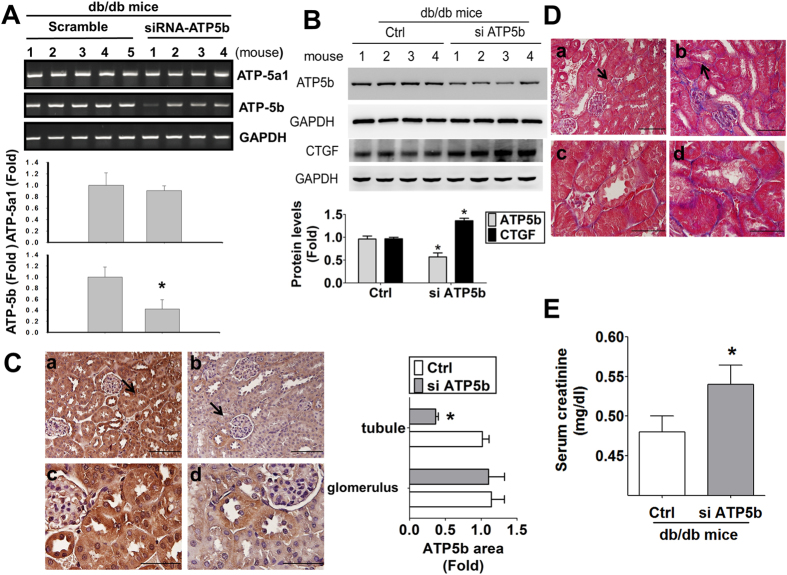
ATP5b siRNA *in vivo* injection increased renal fibrosis and serum creatinine in the kidneys of *db/db* mice. (**A**) ATP5b and homologous member ATP5a1 mRNA detection. The mRNA expressions of ATP5b and ATP5a1 in the kidneys of diabetic *db/db* mice were determined by real-time PCR. Data are presented as means ± SEM (n ≥ 4). *P < 0.05, scramble control group versus ATP5b siRNA group. (**B**) The protein expressions of ATP5b and CTGF were determined by Western blotting. Four representative mice per group were shown. Protein levels of immunoblotting were quantificated by densitometry and normalized by GAPDH levels. Data were presented as means ± SEM (n = 8). *P < 0.05, siRNA versus control (scramble). (**C**) Immunohistochemical staining for ATP5b in the kidneys of *db/db* diabetic mice with *in vivo* siRNA delivery. a and c: control (scramble), b and d: ATP5b siRNA; a and b: scale bar = 100 μm; c and d: scale bar = 50 μm. The arrow showed that indicated areas have enlarged scales in (**C-c**) and (**C-d**). The semi-quantitative assessment of immunohistochemistry with three random areas per section was determined by ImageJ software. (**D**) Masson’s Trichrome staining detected the renal fibrosis in siATP5b injected diabetic mice. The arrow showed that indicated areas have enlarged scales in (**D-c**) and (**D-d**). a and c: control (scramble), b and d: ATP5b siRNA; a and b: scale bar = 100 μm; c and d: scale bar = 50 μm. (**E**) The serum creatinine levels in *db/db* diabetic mice with *in vivo* siRNA delivery. Data are presented as means ± SEM (n = 6). *P < 0.05, siATP5b versus control (scramble).

To confirm the *in vitro* results, ATP5b siRNA *in vivo* delivery was performed in *db/db* mice. The mRNA expression of ATP5b, but not its homologous member ATP5a1, in the kidneys of db/db mice transfected with ATP5b siRNA was significantly decreased (Fig. 9A). The protein expression (Fig. 9B) and immunohistochemical staining (Fig. 9C) of ATP5b were obviously decreased (by about 50%) and CTGF protein expression (Fig. 9B) was significantly increased in the kidneys of *db/db* mice with siRNA *in vivo* delivery. The renal fibrosis (Fig. 9D) and serum creatinine levels (Fig. 9E) were markedly enhanced in *db/db* mice with ATP5b siRNA delivery as compared with scramble control.

### Effects of high glucose on ATP5b expression, ATP levels, mitochondrial membrane potential, and oxidative stress

**Figure 10:**
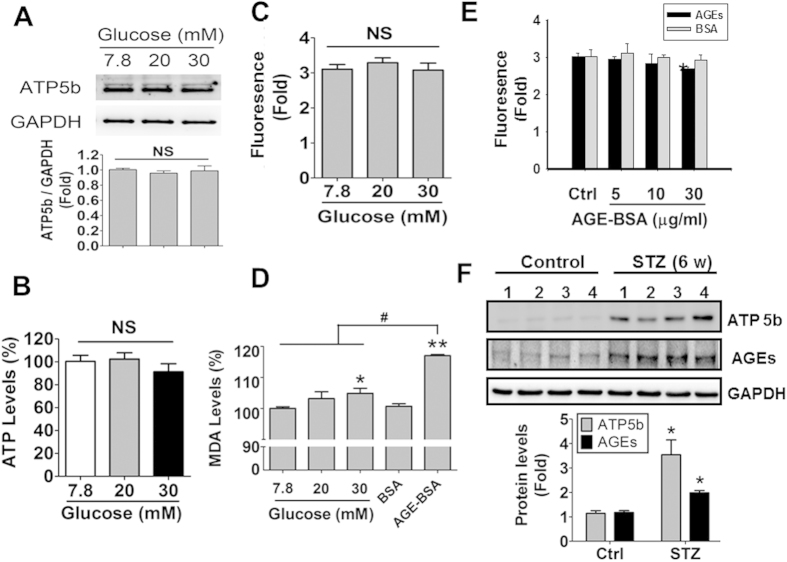
The effects of high glucose on ATP5b protein expression, ATP content, mitochondrial membrane potential and oxidative stress in HK-2 cells. (**A**–**D**) HK-2 cells were cultured in medium containing 7.8, 20, and 30 mM glucose for 24 hours. The ATP5b protein expression (**A**), ATP content (**B**), and mitochondrial membrane potential (**C**), and oxidative stress (MDA level) (**D**) were measured. The protein expressions of ATP5b were measured by Western blotting (**A**). Protein levels were quantified by densitometry and normalized by GAPDH levels. The luminescence ATP detection assay was performed for measuring ATP contents in HK-2 cells (**B**). The fluorescence signaling was detected for mitochondrial membrane potential assay (**C**). Data are presented as means ± SEM (n = 5). NS: non-significant. (**D**) The induction of oxidative stress was measured in HK-2 cells treated with high glucose- or AGE-BSA (10 μg/ml) for 24 hours. (**E**) The effect of AGE-BSA on mitochondrial membrane potential in HK-2 cells. Cells were treated with AGE-BSA (5, 10, and 30 μg/ml) for 24 hours. Data are presented as means ± SEM (n = 5). *P < 0.05, AGE-BSA versus BSA. In some experiments, the protein expression of ATP5b and AGEs in the kidneys of streptozotocin (STZ)-induced diabetic mice (type 1 diabetic model) were detected by Western blotting (**F**). Protein levels were quantified by densitometry and normalized by GAPDH levels. Data are presented as means ± SEM (n = 4). *P < 0.05, STZ versus control.

In order to clarify the effects of high glucose condition, the different concentrations of glucose (7.8, 20, and 30 mM) in culture medium were performed in HK-2 cells. As shown in Fig. 10A–C, ATP5b protein expression, ATP content, and mitochondrial membrane potential were no significant changes in HK-2 cells treated with high glucose. Moreover, both high glucose (30 mM) and AGE-BSA (10 μg/ml) significantly increased lipid peroxidation product MDA levels in HK-2 cells (Fig. 10D); the degree of increased oxidative stress induced by AGE-BSA was more marked than that of high glucose. However, AGE-BSA (30 μg/ml) slightly but significantly decreased the mitochondrial membrane potential in HK-2 cells (Fig. 10E).

We also investigated the protein expression of ATP5b and AGEs in the kidneys of streptozotocin-induced diabetic mice (type 1 diabetic model). Similar to the results in *db/db* mice, both ATP5b and AGEs were overexpressed in the kidneys of streptozotocin-induced diabetic mice (Fig. 10F).

## Discussion

Diabetes is known as a leading cause of kidney disease, which usually progresses over a period of 10–15 years^21^,^25^,^26^. Both glomeruli and tubules are involved in the pathogenesis and pathophysiology of diabetic nephropathy^27^,^28^. The course of development of glomerular pathology in the *db/db* diabetic mice has been suggested to resemble that occurs in the diabetic patients^29^. The *db/db* mice at 12–18 weeks of age have been observed to show the early diabetic nephropathy, causing various degrees of glomerular and tubular injuries and renal fibrosis, as compared with their non-diabetic littermate control *db/m*^+^ mice^29^,^30^. Moreover, sustained exposure to hyperglycemia accelerated the formation of AGEs in many tissues of diabetic patients, which may correlate with the pathological features of diabetes and its complications^31^,^32^. AGEs-RAGE axis has been suggested to be involved in the pathogenesis of diabetic nephropathy^21^. RAGE is a signal transduction receptor for AGEs that can mediate the inflammatory and fibrotic reactions evoked by AGEs in vascular disease and diabetes^33^. AGEs have been shown to induce CTGF expression in tubular epithelial cells via the TGF-β-independent RAGE-ERK/p38-Smad3 cross-talk pathway^16^. Increased AGEs levels and their deposition in the kidneys, which correlate with the development of diabetic nephropathy, have been observed in streptozotocin-induced hyperglycemic rats^34^ and *db/db* diabetic mice^29^,^35^. In the present study, the obvious histopathological changes in glomeruli and glomerular and tubule-interstitial fibrosis were observed in the kidneys of *db/db* mice at 12 weeks of age. The serum creatinine levels in *db/db* mice were markedly increased, which reflected the glomerular damage^29^. AGEs were also observed to deposit in the renal glomerular and tubular areas of *db/db* mice. Nevertheless, there are far less fibrosis and AGEs deposition in the kidneys of *db/m*^+^ mice. We also found that AGEs could significantly increase the protein expressions of α-SMA, fibronectin, CTGF, and collagen-1 in renal proximal tubular HK-2 and LLC-PK1 cells. Therefore, these results indicate that AGEs may play an important role in early diabetic nephropathy in a type 2 diabetic mouse model, which are consistent with the findings of previous studies.

ATP5b belongs to the mitochondrial ATPase complex catalytic core to produce ATP and plays an important role in maintaining the energy homeostasis in the cells. The pancreatic islets isolated from rats with ATP synthase mutation has been found to exhibit the increased oxygen radical formation, decreased ATP content, and impaired β-cell insulin secretion^36^. Recently, ATP synthase system (including ATP5b) and energy balance have been shown to be altered in several pathophysiological conditions such as muscles in obesity and diabetic patients^20^, acute kidney injury^37^, heart in pre-diabetic state^38^, reduction of DAPIT (diabetes-associated protein in insulin-sensitive tissue)^39^, human colon epithelial aging^40^, and hepatic ischemia/reperfusion injury^41^. Xu and colleagues have suggested that ATP5b plays a protective role in ischemic preconditioning against hepatic ischemia/reperfusion injury in mice^41^. Wu and colleagues have also indicated that ATP5b is down-regulated during the aristolochic acid-induced renal tubular atrophy and interstitial fibrosis in rats^42^. Moreover, several pharmacological agents such as mammalian sirtuin 1 (SIRT1) activator SRT1720, 5-HT_2_ receptor agonists, and isoflavones have been shown to promote the mitochondrial biogenesis as indicated by increased expression of ATP5b and other mitochondrial proteins and elevated mitochondrial respiration rate and ATP content in renal proximal tubule cells, protecting tubular cells against oxidant injury or mitochondrial dysfunction^43^,^44^,^45^. The role of ATP5b in AGEs-related diabetic renal fibrosis remains unclear. In the present study, the results of immunohistochemical staining and immunoblotting indicated that ATP5b protein expression in renal tubular areas of *db/db* mice was markedly higher than that in *db/m*^+^ mice. AGEs could also markedly and dose-dependently increase the protein expression of ATP5b in renal tubular HK-2 and LLC-PK1 cells. Gene silencing for ATP5b with siRNA enhanced the expressions of fibrotic signaling molecules α-SMA and CTGF and CTGF promoter activity in AGEs-treated renal proximal tubular cells. In addition, RNA interference technique is not only useful in *in vitro*, but also *in vivo* research for the past few years^46^. We further used ATP5b siRNA to down-regulate ATP5b expression in the kidneys of *db/db* mice and found that renal fibrosis and serum creatinine levels were markedly enhanced. These results suggest that ATP5b may play a protective role in AGEs-related renal fibrosis.

Over-generation of intracellular ROS has been shown to be involved in the inhibition of *de novo* protein synthesis and the stimulation of TGF-β expression in AGEs-treated renal proximal tubular cells^47^. Previous studies have also indicated that BSA induces ER stress and apoptosis through ROS production in renal proximal tubular cells and podocytes^48^,^49^. Nevertheless, more severe oxidative stress causes ATP depletion, necrosis induction, and cell death in oxidant-injured endothelial cells^50^. SIRT1 activator SRT1720 has been found to be capable of accelerating the recovery of mitochondrial functions after acute oxidant injury in renal proximal tubule cells^43^. The present study observed that oxidative stress was induced in diabetic kidneys. Both ATP5b and CTGF were up-regulated after ROS induction in AGEs-treated HK-2 cells, which could be reversed by antioxidant NAC. It may be considered that AGEs induce ROS production to stimulate ATP biosynthesis by ATP synthase for ROS-increased ATP consumption. Therefore, these findings suggest that oxidative stress is involved in the ATP5b regulation and fibrogenesis in renal proximal tubular cells during AGEs exposure.

In this study, the protein expression of ATP5b in renal glomeruli was no significant difference between *db/m*^+^ and *db/db* mice, even though there was remarkable expression of AGEs and fibrosis in the renal glomerular areas of *db/db* mice. AGEs also did not affect the expression of ATP5b, but significantly increased CTGF expression, in cultured mouse and human glomerular mesangial cells. These results suggest that ATP5b may be not involved in the AGEs-induced fibrogenesis in renal glomeruli. In addition, the proximal tubule of nephron reabsorbs more than 60% of the glomerular filtrates^51^. However, comparing with the renal glomerular filtration, the reabsorption in proximal tubule is an energy consuming process^49^. The most common drive for the reabsorption is the basolateral-located Na^+^/K^+^-ATPase and H^+^ pump under the hydrolysis of ATP^52^,^53^. Therefore, the function of renal tubule is preserved by the sufficient ATP synthesis. Previous reports showed that ATP depletion was increased in renal tubule exposed to toxic materials such as fullerenol and 2,3,5-Tris(glutathione-S-yl)-hydroquinone^54^,^55^. *Bothrops alternatus* venom has also been found to significantly induce the functional renal alterations with increased Na^+^/K^+^-ATPase expression and activity *in vivo*^56^. Proteomic approaches have been performed to evaluate the nephrotoxicity of cisplatin on HK-2 cells and found that the expression of ATP synthase subunit α was increased in HK-2 cells exposed to cisplatin^57^. In the present study, we found that the ATP5b expressions in renal tubule cells were increased during AGEs exposure, which may reflect the demand of ATP synthesis to preserve the function of renal tubules in diabetic mice. Therefore, the different energy demand between renal tubule and glomerulus may affect the up-regulation of ATP5b during toxic AGEs accumulation.

In conclusion, in this study, we demonstrate for the first time that ATP5b signaling is involved in the AGEs-related diabetic nephropathy. We found that AGEs formation, ATP5b expression, and histopathological changes and fibrosis were markedly increased in the renal tubular areas of *db/db* diabetic mice. AGEs effectively increased the expression of ATP5b and fibrotic signaling molecules and CTGF promoter activity in cultured renal proximal tubular cells, but not glomerular mesangial cells. We further observed that AGEs/RAGE interaction induced ROS production and increased ATP5b and fibrotic signaling molecules expressions in human renal tubular cells. Silence of ATP5b signaling with siRNA significantly enhanced the expression of AGEs-induced fibrotic signaling molecules and CTGF promoter activity in human renal proximal tubular cells. Finally, ATP5b siRNA *in vivo* delivery significantly augmented renal fibrosis and serum creatinine levels in *db/db* mice. These findings suggest that the increased ATP5b plays an important adaptive or protective role in decreasing the rate of AGEs-induced renal fibrosis during diabetic condition, which may give evidence for developing new therapeutic approaches against the progression of renal fibrosis in early stage of diabetes.

## Methods

### Animals

Male 12-week-old C57BL/6J *db/db* (diabetic littermate) mice and C57BL/6J *db/m*^+^ (non-diabetic littermate control) mice were used in animal experiments. The *db/db* and *db/m*^+^ mice were purchased from Jackson Laboratories (Bar Harbor). For type 1 diabetic mouse model, 6–8 weeks ICR mice were obtained from the Animal Center of the College of Medicine, National Taiwan University (Taipei, Taiwan). Mice were fasting overnight and then injected intraperitoneally with a single dose of 100 mg/kg streptozotocin (STZ, Sigma-Aldrich) to mimic diabetic condition. After STZ injection for one week, the blood glucose level was over than 400 mg/dl. Six weeks later, the kidneys from 6 mice were carefully isolated for immunoblotting detection. All animal studies were approved by the ethical review committee of National Taiwan University, College of Medicine and were carried out in accordance with regulations of Taiwan and NIH guidelines on the care and welfare of laboratory animals. All animals were treated humanely and with regard for alleviation of suffering.

### Biochemical measurement

Serum biochemical parameters such as serum glucose, insulin, HbAlc, and creatinine were determined by a commercially available clinical chemistry analyzer (Roche). Serum AGEs levels were measured by a mouse ELISA kit (Biotang).

### Histology and immunohistochemistry

The 4-μm-thick paraffin-embedded renal tissue sections were stained with Masson’s trichrome for detection of fibrosis. For immunohistochemistry, the primary antibodies for AGEs (ab23720, Abcam) and ATP5b (sc-55597, Santa Cruz Biotechnology) were used. For anti-AGE antibody (ab23720, Abcam), the immunogen is Advanced Glycation End Products-a mixture of BSA-AGE and human serum albumin (HAS)-AGE. The epitope is a modification and not species specific; the antibody was tested in human but should cross react with all species. In some experiments, the kidney samples of type 2 diabetic patients or normal subjects were derived from total nephrectomies or biopsies of donor kidneys for transplantation, respectively, with written approval from the institutional Ethics Committee at National Taiwan University Hospital, Taipei, Taiwan and with informed consent from patients. Images of the sections were obtained using a biological microscope Olympus DP70 (Olympus, Tokyo, Japan) and DP controller digital imaging software for research (Olympus) under 400 or 1000 magnification.

### Quantification of protein expression in immunohistochemistry

For quantitative immunohistochemistry, the levels of protein expressions were analyzed through a blinded fashion with a digital image analysis software (ImageJ version 1.48, National Institutes of Health, USA)^58^.

### Double immunofluorescence staining

The 4-μm-thick paraffin-embedded renal tissue sections of *db/m*^+^ or *db/db* mice were deparaffinized with xylene and rehydration by 90%, 75%, and 50% alcohol for 5 min each. Subsequently, sections were retrieving in the microwave oven and cover with citrate buffer (pH 6.0) for 30 min. After cooling and rinse in PBST (115 mM NaCl, 3.6 mM KCl, 1.3 mM KH_2_PO_4_, 25 mM NaHCO_3_, and 0.05% tween 20, pH 7.4), sections were incubated with primary antibodies ATP5b (1:50; sc-55597, Santa Cruz Biotechnology) and AQP-1^59^ (1:40; Abcam) overnight. The sections were then washed with PBST buffer for 3 times and treated with anti-mouse FITC or anti-rabbit TRITC fluorescent secondary antibodies (1:500; 1:250, Sigma-Aldrich) for 1 hour. Finally, Hoechst 33258 (1 μg/ml, Sigma-Aldrich) counter stain was performed. The sections were washed and mounted with Mounting medium (Dako Inc, Carpinteria, CA, USA). The samples were observed and evaluated at 15 visual random fields on × 200 magnification.

### AGE-BSA preparation

AGE-BSA was prepared as previously described^22^. Briefly, BSA (25 mg/ml) was incubated under sterile conditions with 0.1 M glyceraldehyde in 0.2 M NaPO_4_ buffer (pH 7.4) for 7 days. The unincorporated sugars were then removed by PD-10 column chromatography and dialysis against phosphate-buffered saline, and then filter-sterilized using a 0.22 μm Millipore filter (Millipore). Controlling non-glycated BSA was incubated in the same conditions except for the absence of reducing sugars. Prepared samples were tested for endotoxin using Endospecy ES-20S system (Seikagaku, Tokyo, Japan). AGE-BSA was identified using a MALDI-TOF/TOF mass spectrometry (UltraFlexIII, Bruker, Billerica, MA, USA). The components of AGEs include carboxymethyllysine (CML), carboxyethyllysine (CEL), pentosidine, and others^7^,^8^,^9^. The major component of AGEs is CML.

### Cell lines and primary cells

Porcine renal proximal tubular cell line (LLC-PK1) and human renal proximal tubular cell line (HK-2) and mouse glomerular mesangial cell line (SV40 MES 13) were purchased from the American Type Culture Collection. The primary human renal mesangial cells (Catalog#4200) and primary human renal proximal tubular cells (Catalog#4100) were purchased from ScienCell Research Laboratories (Carlsbad, CA, USA). Cell passage and culture conditions were followed as manufacturer’s procedures. All culture reagents and media were acquired from Gibco. These cells were incubated at 37 °C and 5% CO_2_.

### Cell viability assay

Cell viability was determined by cell counting kit-8 (CCK-8, #96992, Sigma-Aldrich) assay. Cells (1 × 10^4^) were seeded in 96 well plates at 37 °C and 5% CO_2_ overnight. Subsequently, the cells were treated with tested materials for 24 hours, and then 10 μl of CCK-8 solution was added to each well. After 3 hours incubation, the absorbance was measured at 450 nm and 650 nm using a microplate reader (Bio-Rad).

### Immunoblotting

The detection of protein expression in cells and renal tissues was performed by Western blotting as described previously^60^. The protein samples were separated by SDS-PAGE and transferred onto the Immobilon P membranes (Millipore). After blocking with for 5% skim milk solution for 2 hours, the membranes were incubated overnight at 4 °C with primary antibodies for AGEs (#KH001, TransGenic Inc.), α-SMA (#A5228, Sigma-Aldrich), CTGF (#C4871, Sigma-Aldrich), collagen I (Abcam), ATP5b (#sc-55597, Santa Cruz Biotechnology), Fibronectin (Abcam), collagen IV (#C1926, Sigma-Aldrich), Fibrinogen γ (Novus Biologicals) and RAGE (#R5278, Sigma-Aldrich). Follow, the secondary antibodies were incubated for 1 hour and the membranes were detected by using enhanced chemiluminescence (Thermo Fisher Scientific) on Fuji Film LAS-4000 mini performing system. The blotting bands were quantified by densitometric analysis using Multi Gauge v3.2 software.

### Blockade of RAGE-ligand interaction assay

To assess the interaction between RAGE antibody (#Mab11451, R&D systems) and RAGE protein, coimmunoprecipitation (co-IP) was performed. Firstly, the RAGE antibody was labeled with a fluorescence material (FITC). HK-2 cells (1 × 10^6^) were treated with FITC-labeled RAGE antibody (10 μg/ml) for 1 h at 4 °C, and then HK-2 cells were treated with AGE-BSA (10 μg/ml) for 24 hours. Next, the cells were harvested and lysed on ice for 30 min with lysis buffer, containing 150 mM NaCl, 0.5% sodium deoxycholate, 0.1% TX-100, 50 mM Tris, pH 8.0, and protease inhibitors cocktail. Cell debris was removed by centrifugation at 14,000 rpm for 10 min at 4 °C. The supernatant of lysate was incubated with or without biotin-conjugated-RAGE polyclonal antibody (2 μg/ml, #bs-4999R-Biotin, Bioss) in the presence of streptavidin-conjugated agarose beads (#S1638, Sigma-Aldrich) at 4 °C overnight. The immunoprecipitates were washed 5 times with washing buffer (150 mM NaCl, 20 mM Tris, pH7.4, 0.1% TX-100) and detected the fluorescent signaling by using a ELISA reader.

### RNA interference

The ATP5b short interfering RNA (siRNA) and scramble control were purchased from Invitrogen. HK-2 cells were seeded to a confluency of 80% on 6-well plates the day before transfection. Subsequently, the cells were transiently transfected with scramble or ATP5b siRNA using Lipofectamine RNAiMAX Reagent (Invitrogen) according to the manufacturer’s instructions. The siRNA/Lipofectamine RNAiMAX complexes were removed after 6.5 hours and the cells were incubated at 37 °C. After 24 hours, the transfected cells were treated with AGE-BSA and non-glycated BSA at the designated concentration and for the indicated time course.

### Real-time RT-PCR

HK-2 cells were treated with tested materials for 24 hours. Subsequently, cells were collected and detected the mRNA expression by Bio-Rad iQ5 Real-time RT-PCR Detection System. Briefly, Total RNA (5 μg) was reverse transcribed in a reaction volume of 30 μl using the Promega reverse transcriptase reagent mix. 100 ng RT products were used as template for amplification using the SYBR Green PCR amplification reagent (Qiagen) and gene-specific primers. The amount of DNA was normalized by the 18S rRNA and GAPDH signals amplified in one separate reaction. The primer sets of human CTGF (forward: TTAGCGTGCTCACTGACCTG; reverse: GCCACAAGCTGTCCAGTCTA), TGF-β (forward: TGGAAACCCACAACGAAATCTATG; reverse: GTTGCTGTATTTCTGGTACAGCTC), 18S (Forward: AGTCCCTGCCCTTTGTACACA; Reverse: CGATCCGAGGGCCTCACTA), and GAPDH (Forward: TGGCACAGTCAAGGCTGAGA; Reverse: CTTCTGAGTGGCAGTGATGG) were used. In some experiments, the mRNA expressions of ATP5b and ATP5a1 in the kidneys of diabetic *db/db* mice were detected. The primer sets of mouse ATP5b (Forward: TTCAGGGGCACCAATCAAAATTC; Reverse: CAACCTTTATCCCAGTCACCAGA) and mouse ATP5a1 (Forward: AATGTTCAAGCAGAGGAGATGGT; Reverse: TCCATCAATAGCATTACCGAGGG). The mRNA expression was detected by the iQ5 optical system software ver.2 (Bio-Rad).

### Luciferase reporter assay

The full-length CTGF plasmid DNA (pCTGF) was a kind gift from Dr. Min-Liang Kuo (National Taiwan University, Taipei, Taiwan). The plasmid sequences were confirmed by DNA sequencing. HK-2 cells were grown to 90% confluence in a 12-well plates and transiently co-transfected with a mixture of 0.75 μg pCTGF reporter and 0.75 μg Renilla vector using DNA transfection reagent (SignaGen Laboratories) according to the manufacturer’s instruction. Luciferase reporter assay was performed by a Dual Luciferase Reporter Kit (Promega BioSciences). Firefly and Renilla luciferase activities were assessed by luminescent reader Mithras LB940 (Berthold Technologies).

### Measurement of oxidative stress in the kidneys

The renal tissue lysates were centrifuged at 12,000 × g for 15 minutes. The supernatants were collected for detecting the levels of H_2_O_2_ and lipid peroxidation determined by H_2_O_2_ assay kit (BioVision) and thiobarbituric acid reactive substance assay kit (Cayman chemical), respectively. For H_2_O_2_ assay, the samples were measured at colorimetrical wavelength of 570 nm using a micro-plate reader. For lipid peroxidation assay, the samples were determined by reference to a concentration of malondialdehyde standard curve at colorimetrical wavelength of 530–540 nm.

### ROS detection

HK-2 cells were treated with tested materials for 24 hours. Media were removed and washed with PBS and then incubated with 20 nM DCFDA for 30 minutes. The cells were harvested for flow cytometric analysis by FACSCalibur Flow Cytometer (BD Bioscience).

### ATP content

Measurement of cellular ATP content was performed by luminescence ATP detection assay system (Perkin Elmer). Cells (1×10^4^) were cultured in 96 well plates at 37 °C and 5% CO_2_. After 24 hours pre-incubation, each well was treated with tested materials for 24 hours and then ATP assay reagent was added to each well. After 10 minutes, the samples were handpicked into white 96-microplate for luminescence measurement. For tissue ATP content assay, the tissues were lysed and removed the tissue debris. Then, the tissue lysis was diluted 10 × and added ATP assay reagent for luminescence measurement.

### Delivering siRNA *in vivo*

Deprotected and annealed ATP5b or scrambled siRNAs were dissolved in RNasefree suspension buffer. Sixteen male 12-week-old *db/db* mice were randomized into two groups (control and siRNA). The siRNA mice received 100 μl of Invivofectamine® 3.0 Reagent (Life Technologies) in combination with 40 μg ATP5b siRNA. Control animals received 100 μl of Invivofectamine® 3.0 Reagent reagent in combination with 40 μg scramble siRNA. Diabetic mice were rapidly injected scramble or ATP5b siRNA through the lateral tail veins, once per 3 days for a total of 4 times. All mice were sacrificed 48 h after last injection. The kidneys were collected for Western blot, Masson’s trichrome staining, and immunohistochemistry assay. The serum was harvested for creatinine assay.

### Mitochondrial membrane potential assay

The assay protocol was according to the manufacturer’s instructions (Cayman chemical company). Briefly, 5 × 10^4^ HK-2 cells were seeding in the 96-well black plate with complete culture medium. After stable for 24 hours, HK-2 cells were refreshed with complete medium and then treated with glucose (up to 20 and 30 mM) and AGE-BSA (0, 5, 10, 30, 50 μg/ml) for 24 hours. Subsequently, total medium were removed and substituted by the JC-1 staining solution for 30 min. Remove staining solution again and then wash, centrifuge (400 × g) for two times. Finally, the mitochondrial membrane potential was detected with the excitation and emission at 535 nm and 595 nm for healthy cells (J-aggregates) and with the excitation and emission at 485 nm and 535 nm for unhealthy cells (J-monomers), respectively. The plate was detected by a Synergy^TM^ 2 multi-detection microplate reader (BioTek, Tokyo. Japan).

### Statistical analysis

Data are expressed as means ± SEM. The significant difference from the respective controls for each experimental test condition was assessed by one-way analysis of variance and Dunnett’s test. The difference is significant if the *P*-value is less than 0.05. Statistical analysis was performed using GraphPad Prism V5.01 software.

## Additional Information

**How to cite this article**: Guan, S.-S. *et al.* ATP synthase subunit-ß down-regulation aggravates diabetic nephropathy. *Sci. Rep.* **5**, 14561; doi: 10.1038/srep14561 (2015).
